# The use of the rapid osmotic fragility test as an additional test to diagnose canine immune-mediated haemolytic anaemia

**DOI:** 10.1186/1751-0147-55-74

**Published:** 2013-10-25

**Authors:** Geert Paes, Dominique Paepe, Evelyne Meyer, Annemarie T Kristensen, Luc Duchateau, Miguel Campos, Sylvie Daminet

**Affiliations:** 1 Department of Small Animal Medicine and Clinical Biology, Faculty of Veterinary Medicine, University of Ghent, Salisburylaan 133, 9820 Merelbeke, Belgium; 2 Department of Pharmacology, Toxicology and Biochemistry, Faculty of Veterinary Medicine, University of Ghent, Salisburylaan 133, 9820 Merelbeke, Belgium; 3 Department of Small Animal Clinical Sciences, Faculty of Health and Medical Sciences, University of Copenhagen, Dyrlægevej 16, DK-1870 Frederiksberg C, Denmark

**Keywords:** Osmotic fragility, Canine immune-mediated haemolytic anaemia, Hyperlipidemia

## Abstract

**Background:**

Diagnosing canine immune-mediated haemolytic anaemia (IMHA) is often challenging because all currently available tests have their limitations. Dogs with IMHA often have an increased erythrocyte osmotic fragility (OF), a characteristic that is sometimes used in the diagnosis of IMHA. Since the classic osmotic fragility test (COFT) is time-consuming and requires specialized equipment, an easy and less labour-intensive rapid osmotic fragility test (ROFT) has been used in some countries, but its diagnostic value has not yet been investigated.

This study aimed to evaluate erythrocyte osmotic fragility in dogs with and without IMHA, to compare results of the classic (COFT) and rapid (ROFT) test and to assess the value of the ROFT as diagnostic test for canine IMHA.

Nineteen dogs with IMHA (group 1a), 21 anaemic dogs without IMHA (group 1b), 8 dogs with microcytosis (group 2), 13 hyperlipemic dogs (group 3), 10 dogs with lymphoma (group 4), 8 dogs with an infection (group 5) and 13 healthy dogs (group 6) were included.

In all dogs, blood smear examination, in-saline auto-agglutination test, Coombs’ test, COFT and ROFT were performed. In the COFT, OF5, OF50 and OF90 were defined as the NaCl concentrations at which respectively 5, 50 and 90% of erythrocytes were haemolysed.

**Results:**

Compared with healthy dogs, OF5 and OF50 were significantly higher in group 1a (*P* < 0.001) and OF5 was significantly higher in group 3 (*P* = 0.0266). The ROFT was positive in 17 dogs with IMHA, 10 hyperlipemic dogs, one anaemic dog without IMHA and one healthy dog.

**Conclusions:**

Osmotic fragility was increased in the majority of dogs with IMHA and in dogs with hyperlipidemia, but not in dogs with microcytosis, lymphoma or an infection. Although more detailed information was obtained about the osmotic fragility by using the COFT, the COFT and ROFT gave similar results. The ROFT does not require specialized equipment, is rapid and easy to perform and can be used easily in daily practice. Although, the ROFT cannot replace other diagnostic tests, it may be a valuable additional tool to diagnose canine IMHA.

## Background

The diagnosis of canine immune-mediated haemolytic anaemia (IMHA) can be challenging as there is currently no ‘gold standard’ [1,2]. Therefore, a diagnosis of IMHA in dogs is clinically made based upon the presence of haemolytic anaemia in combination with evidence of immune-mediated destruction (marked spherocytosis and/or a positive Coombs’ test or in-saline auto-agglutination test) [1,3,4]. Spherocytosis is a strong indicator of IMHA, but identification of spherocytes on blood smears is subjective and can be difficult for practitioners that do not evaluate blood smears on a daily basis [5]. Some authors suggest that when placed in a hypotonic solution, spherocytes haemolyse at higher NaCl concentrations than normal red blood cells (RBCs), because of their lower volume to surface ratio [5,6]. This may result in an increased osmotic fragility (OF) [4]. Two protocols to evaluate the OF have been described in dogs [5,6]. In the first, which we named the classical OF test or the COFT, 15 different NaCl concentrations are used to determine at which concentrations haemolysis is initiated and subsequently completed [6]. In the second, which we named the rapid OF test or the ROFT, OF is estimated semi-quantitatively using only two NaCl concentrations [5]. Since the COFT is time-consuming and requires specialized equipment, it is not applicable for use in daily practice. Therefore, the easy and less labour-intensive ROFT, could be an appealing alternative. The ROFT has been used often in the diagnostic work up of canine IMHA in Belgium and the Netherlands, but its diagnostic value has never been evaluated [4,6]. Furthermore, the literature about the influence of other systemic diseases or pathologic conditions on OF is limited.

Increased RBC OF has already been reported as a feature of certain hereditary erythrocyte membrane defects [7-15], microcytosis due to intestinal parasitism [16,17], intoxications with β-acetylphenylhydrazin [18] and hyperlipidemia [19]. An increase in OF, resulting in anaemia, has been observed in rabbits, rats, mice, guinea pigs and dogs with experimentally induced hypercholesterolemia [19,20]. Although it has yet to be described in dogs, hypertriglyceridemia can have similar effects on RBCs [21,22]. Finally, severe liver insufficiency can induce spur cell development with increased OF [19]. There are no published studies evaluating OF in dogs with natural occurring hyperlipidemia, neoplasia or infections.

The present study aimed to evaluate OF in anaemic dogs with and without IMHA and in dogs with certain specific clinical conditions for which an increased osmotic fragility has already been described in human studies or in experimental studies. Furthermore, results of the COFT and ROFT were compared to evaluate if the ROFT could be used as an alternative test for the labour-intensive COFT. Finally, by calculating the sensitivity and specificity of the ROFT in anaemic dogs we aimed to assess the value of the ROFT as additional test to diagnose canine IMHA.

## Methods

A prospective clinical study was conducted between January 2007 and August 2009.

### Animals

All patients (mostly referral cases) were recruited at the Faculty of Veterinary Medicine, Small Animal Clinic of Ghent University.

Dogs with a moderate to severe anaemia (group 1) were included if they had a packed cell volume (PCV) prior to infusion therapy ≤ 30%, (reference interval: 43–59%) and were divided into group 1a (dogs with IMHA) and group 1b (dogs with anaemia due to conditions other than IMHA). Diagnosis of IMHA was based on the presence of haemolytic anaemia (hyperbilirubinemia, haemoglobinaemia, bilirubinuria, haemoglobinuria) in combination with either a moderate to marked spherocytosis or a positive Coombs’ or in-saline auto-agglutination test. Dogs with intravascular haemolysis, characterized by the presence of haemoglobinaemia and/or haemoglobinuria that may or may not be combined with hyperbilirubinemia and bilirubinuria as well as dogs with extravascular haemolysis, characterized by hyperbilirubinemia and/or bilirubinuria in absence of haemoglobinaemia and haemoglobinuria, were included. In every dog diagnosed with IMHA, further diagnostic examinations such as urinalysis, thoracic radiographs, abdominal ultrasonography and, depending on the patient history (travel history, geography, seasonality), serology and/or PCR for blood parasites and leptospirosis was performed to exclude an underlying disease such as neoplasia, an infection or the presence of a metallic foreign body. Dogs were considered having primary IMHA if medical imaging studies did not reveal any significant abnormalities and serology and/or PCR tests for blood parasites or other infections were negative. Group 1a and 1b dogs were considered having a regenerative anaemia if they had an absolute reticulocyte count over 60 000/μl. Dogs with microcytosis (group 2), anaemic and non-anaemic, were included if they had a RBC mean corpuscular volume (MCV) of ≤ 60 fl (reference interval: 63–77 fl). Breeds predisposed to microcytosis, such as Shiba Inu and Akita Inu, were excluded [23]. Dogs with hyperlipidemia (group 3) were included if they had a fasted (minimum 12 hours) serum cholesterol (Chol) concentration of ≥ 12 mmol/l (reference interval: 3.33-10.21 mmol/l) and/or triglyceride (TG) concentration ≥ 2 mmol/l (reference interval: 0.02 – 1.86 mmol/l). Dogs with multicentric lymphoma (group 4) were included based on compatible clinical signs and cytology or histopathology confirmed diagnosis. Dogs that were diagnosed with an infection (either parasitic, mycotic, viral or bacterial) (group 5) joined the study if they showed compatible clinical signs in combination with positive bacteriology, serology and/or PCR results. Group 1–5 dogs that received medication for longer than 3 days prior to admission and those for whom a definitive diagnosis could not be made, were excluded. For group 1–5 there were no age restrictions. Furthermore, except for group 2, all dog breeds were considered for inclusion. The results of the patient groups were compared to those of healthy, client-owned dogs (group 6), older than 6 months, that had no significant abnormalities on physical examination, complete blood count (CBC) and serum biochemistry, and did not receive any medication for two months prior to enrolment. For the healthy dogs, we specifically selected dogs that did not belong to breeds, in which hereditary erythrocyte membrane defects associated with increased OF have been described (Alaskan Malamute, Miniature and Middle Schnauzers, Golden Retriever, Dutch Partridge Dog, English Springer Spaniel, American Cocker Spaniel, Whippet, Shetland Sheepdog, German Spaniel and Beagle) [7-15].

All owners were informed about the purposes of the study and signed a written consent to allow inclusion of their dog.

### Laboratory tests

Blood was collected by jugular vein puncture, stored at 4°C and analysed within 24 hours following collection. Serum Chol and TG concentrations were measured in every dog from group 2–6 on a fasted (minimum 12 hours) blood sample. Serum Chol and TG concentrations were only measured in group 1 dogs if the dog had been anorectic for at least 12 hours and if the clinical condition of the dog allowed to take a sufficient amount of blood to perform these measurements. Three blood smears, stained with Diff-Quik (Diff-Quik Fix, Medion Diagnostics, Düdingen, Switzerland), May-Grünwald Giemsa (MGG) (Carl Roth, Karlsruhe, Germany) and New Methylene Blue (Sigma-Aldrich, Steinheim, Germany) were made. The degree of spherocytosis was mainly assessed based on the MGG stained blood smears by counting the number of spherocytes in 10 different oil immersion fields (100×) (HPF), followed by calculating the mean value per HPF. Dogs with <1 spherocyte, 1–3 spherocytes, 3–5 spherocytes and ≥ 5 spherocytes per HPF were defined as having ‘no’, ‘mild’, ‘moderate’ and ‘marked’ spherocytosis, respectively. The degree of spherocytosis was initially determined when the patient was admitted to the clinic by the primary clinician for that specific case. Based on the findings in combination with the other diagnostic tests a diagnosis was reached and treatment was started. Afterwards, the blood smears of all dogs were send off to the Department of Small Animal Clinical Sciences, Faculty of Health and Medical Sciences, University of Copenhagen, Denmark to be evaluated by Dr. A.T. Kristensen that was blinded from all patient information (signalment, group, physical and clinicopathologic abnormalities). Dogs were only included in the study if there was no discordance in the number of spherocytes found by the primary clinician and found by Dr. Kristensen that would have resulted in a different diagnosis for that patient.

The in-saline auto-agglutination test was carried out by mixing one drop of EDTA blood with 4 drops of NaCl 0.9% at room temperature on a smear slide. RBC agglutination was immediately evaluated macroscopically and microscopically [24-26]. A direct Coombs’ test was performed on EDTA blood using the ID-Gel test anti-canine globulin (DiaMed-VET, Morat, Switzerland), with 6 microtubes containing anti-canine globulin (rabbit anti-IgG, anti-IgM and anti-C3) within the gel matrix [27,28]. To perform the test, first a 0.8% red cell suspension was made by gently mixing 10 μl of packed cells with 1.0 mL of ID-diluent VET 2. Of this red cell suspension, 50 μL was added to each microtube of the ID-Gel Test card. After which, the ID-Gel Test card was centrifuged for 10 minutes in the ID-centrifuge. The test was positive when agglutinated cells formed a red line on the surface of the gel or agglutinates dispersed in the gel. The test was negative when the cells lay on the bottom of the microtubes. To avoid interference caused by severe auto-agglutination, the RBCs of the patient were first washed two times in phosphate buffered saline (PBS; pH 7.4). Furthermore, a negative control was included for every Coombs’ test, using negative control cards that contain gel matrix without antibody.

The COFT and ROFT were performed on whole blood with sodium heparin as anticoagulant, the former as described by Jain (1986). Of a 10% PBS solution (pH 7.4) 15 5-mL dilutions, representing NaCl concentrations from 0.85% to 0.10%, were made. A 16^th^ tube contained 5 ml of distilled water. To each tube, 0.02 ml blood was added and mixed by inversion. After standing 30 minutes at room temperature, the tubes were centrifuged for 10 minutes at 795× *g*. The optical density of each supernatant was established spectrophotometrically at 540 nm, using distilled water as a blank. For each dilution the percentage of haemolysis was calculated by assuming the haemolysis in the 16^th^ tube to be a 100%. OF5, OF50 and OF90 were determined as being the NaCl concentration in which respectively 5, 50 and 90 per cent of the RBCs were haemolysed. A derivative and a cumulative fragiligram were thus obtained [6].

For the ROFT, OF was estimated semi-quantitatively using two NaCl solutions, 0.9% and 0.55% [5]. The first tube contained 5 ml of NaCl 0.9% and the second 3 ml of NaCl 0.9% diluted with 2 ml of distilled water. Five drops of the patient’s blood were added to each tube, incubated for 5 minutes at room temperature and centrifuged for 5 minutes at 2431× *g*. The test was considered positive when the supernatant was colourless or yellow (due to hyperbilirubinemia) in the first tube and red in the second tube, and characterized as negative when an obvious colour difference between both tubes was not discernible.

### Statistical analysis

OF5, OF50 or OF90 were normally distributed according to the Shapiro Wilk test; these variables were therefore compared globally between different groups by the *F*-test. All clinical groups were compared with the control group using Dunnett’s adjustment technique to adjust the *P*-value for multiple comparisons. The global significance level was set at 0.05.

Different variables (PCV, MCV, number of spherocytes, Chol, TG) were not normally distributed and were compared between the ROFT positive and ROFT negative dogs and between the Coombs positive and Coombs negative dogs using Wilcoxon rank sum test with a global significance level at 0.05. Furthermore, by using the same test, OF50 was compared between the Coombs’ positive and Coombs’ negative dogs and between the in-saline auto-agglutination positive and negative dogs.

The correlation between the number of spherocytes and the OF50 was determined by calculating the Pearson’s correlation coefficient with a global significance level at 0.001.

For group 1, the number of reticulocytes (log transformed) was compared between ROFT positive and ROFT negative dogs by a *t*-test with a global significance level at 0.05 and, by using the same test, OF5, OF50 and OF90 were compared between dogs with regenerative and dogs with non regenerative anaemia.

The sensitivity and specificity of the ROFT for diagnosing IMHA in anaemic dogs were determined based upon the results from the dogs from group 1a and group 1b with a 95% confidence interval. All statistical tests were performed by using SAS version 9.2.

## Results

### Animals

In total, 92 dogs were included and divided among 6 groups. Signalment data of all dogs are presented in Table 1. Table 2 presents the clinical diagnosis for all dogs.

**Table 1:** Signalment data for group 1a (IMHA), group 1b (anaemia, no IMHA), group 2 (microcytosis), group 3 (hyperlipidemia), group 4 (lymphoma), group 5 (infections) and group 6 (healthy)

**Group**	**Breeds**	**Sex**	**Age (years)**
**1a (n = 19)**	Shih Tzu (n = 3), Belgian Shepherd (n = 3), Kuvasz (n = 1), Maltese Dog (n = 1), Great Dane (n = 1), American Cocker Spaniel (n = 1), Miniature Pincher (n = 1), Rottweiler (n = 1), Border Collie (n = 1), American Staffordshire Terrier (n = 1), Fox Terrier (n = 1), Labrador Retriever (n = 1), Jack Russell Terrier (n = 1), Dutch Partridge Dog (n = 1), mixed breed (n = 1)	F (n = 6)	4.9 ± 2.7 (0.5-10)
		FN (n = 5)	
		M (n = 6)	
		MN (n = 2)	
**1b (n = 21)**	Bernese Mountain Dog (n = 4), Jack Russell Terrier (n = 4), Belgian Shepherd (n = 2), mixed breed (n = 2), Maltese Dog (n = 2), Miniature Schnauzer (n = 2), Beagle (n = 1), Labrador Retriever (n = 1), Miniature Pincher (n = 1), Cavalier King Charles Spaniel (n = 1), Big Münsterländer (n = 1)	F (n = 6)	7.2 ± 4.3 (0.5-14)
		FN (n = 5)	
		M (n = 8)	
		MN (n = 2)	
**2 (n = 8)**	Labrador Retriever (n = 2), Doberman Pincher (n = 1), Jack Russell Terrier (n = 1), Maltese Dog (n = 1), Yorkshire Terrier (n = 1), mixed breed (n = 1), American Staffordshire Terrier (n = 1)	F (n = 2)	8.6 ± 3.5 (1–12)
		FN (n = 2)	
		M (n = 3)	
		MN (n = 1)	
**3 (n = 13)**	Mixed breed (n = 2), Yorkshire Terrier (n = 2), Jack Russell Terrier (n = 1), Boxer (n = 1), Fox Terrier (n = 1), Cavalier King Charles Spaniel (n = 1), Weimaraner (n = 1), English Cocker Spaniel (n = 1), Golden Retriever (n = 1), Newfoundlander (n = 1), Wolfspitz (n = 1)	F (n = 2)	9.3 ± 2.9 (3–13)
		FN (n = 2)	
		M (n = 7)	
		MN (n = 2)	
**4 (n = 10)**	Vizla (n = 1), Bernese Mountain Dog (n = 1), American Cocker Spaniel (n = 1), Fox Hound (n = 1), Dogo Argentino (n = 1), Golden Retriever (n = 1), Border Collie (n = 1), American Staffordshire Terrier (n = 1), Daschound (n = 1), Bouvier des Flandres (n = 1)	F (n = 2)	7.9 ± 2.4 (4–12)
		FN (n = 2)	
		M (n = 4)	
		MN (n = 2)	
**5 (n = 8)**	German Pointer (n = 1), Fila Brasileiro (n = 1), American Staffordshire Terrier (n = 1), Cavalier King Charles Spaniel (n = 1), Chow Chow (n = 1), Labrador Retriever (n = 1), Beauceron (n = 1), mixed breed (n = 1)	F (n = 4)	6.7 ± 3.8 (2–13)
		M (n = 4)	
**6 (n = 13)**	Mixed breed (n = 3), Nova Scotia Duck Tolling Retriever (n = 2), Maltese Dog (n = 2), Labrador Retriever (n = 2), Jack Russell Terrier (n = 1), Boxer (n = 1), Border Collie (n = 1), English Staffordshire Terrier (n = 1)	F (n = 5)	6.1 ± 3.5 (1–11)
		FN (n = 1)	
		M (n = 4)	
		MN (n = 3)	

The data for age are expressed as mean ± standard deviation (range).

F: female; FN: female neutered; M: male; MN: male neutered.

**Table 2:** The clinical diagnosis for group 1a (IMHA), group 1b (anaemia, no IMHA), group 2 (microcytosis), group 3 (hyperlipidemia), group 4 (lymphoma), and group 5 (infections)

**Group**	**Disease category**	**Diseases**
**1a (n = 19)**	Immune-mediated haemolytic anaemia	Primary immune-mediated haemolytic anaemia (n = 17), secondary immune-mediated haemolytic anaemia (n = 2): right hind limb abscess, after Permethrin and Imidacloprid^a^ treatment
**1b (n = 21)**	Anaemia, no immune-mediated haemolytic anaemia	Splenic hemangiosarcoma (n = 5), histiocytic sarcoma (n = 2), disseminated mastocytoma (n = 1), gastro-intestinal bleeding due to long-term NSAID administration (n = 2) and intestinal neoplasia (n = 2), coumarine intoxication (n = 2), methemoglobinemia due to onion intoxication (n = 1), blood loss due to immune-mediated thrombocytopenia (n = 2), chronic kidney disease (n = 1), hypoadrenocorticism (n = 1), factor X deficiency (n = 1), anaemia of inflammatory disease (n = 1)
**2 (n = 8)**	Microcytosis	Iron deficiency due to chronic gastro-intestinal blood loss (n = 6), hypoadrenocorticism (n = 1), portosystemic shunt (n = 1)
**3 (n = 13)**	Hyperlipidemia	Hyperadrenocorticism (n = 7), diabetic ketoacidosis with pancreatitis (n = 1), pancreatitis (n = 1), cholestatic liver disease (n = 1), hypothyroidism (n = 1), nephrotic syndrome (n = 1), primary hyperlipidemia (n = 1)
**4 (n = 10)**	Multicentric Lymphoma	B-cell lymphoma (n = 5), T-cell lymphoma (n = 2), lymphoma with unknown immunophenotype (n = 3)
**5 (n = 8)**	Infectious disease	Pyometra (n = 2), septic peritonitis (n = 2), prostatitis (n = 1), chronic Erlichiosis (n = 1), Leishmaniasis (n = 1), septic pericarditis (n = 1)

^a^Advantix®, Bayer Health Care, Brussels, Belgium.

### Laboratory tests

For all groups, the results of the PCV, MCV, Chol, TG and number of spherocytes on the blood smear are summarized in Table 3. For group 1a the anaemia was regenerative in 14/19 (74%) of the dogs, while it was regenerative in 11/21 (53%) of group 1b dogs. The minimum, maximum and median absolute reticulocyte count were respectively 3000/μl, 249000/μl and 81000/μl for group 1a and 7340/μl, 329260/μl and 100000/μl for group 1b. The dogs from group 1a with ‘no spherocytosis’ were diagnosed with primary IMHA, based on a positive in-saline auto-agglutination (n = 3) and/or Coombs’ test (n = 2) and the absence of an underlying cause for the anaemia. None of the dogs in group 1b, 2, 3 or 5 with mild, moderate or marked spherocytosis, showed signs of haemolysis, and they all had a negative Coombs’ and in-saline auto-agglutination test.

**Table 3:** Results of the packed cell volume, mean corpuscular volume, cholesterol and triglyceride concentration, number of spherocytes, in-saline auto-agglutination and Coombs’ test for group 1a (IMHA), group 1b (anaemia, no IMHA), group 2 (microcytosis), group 3 (hyperlipidemia), group 4 (lymphoma), group 5 (infections) and group 6 (healthy)

		**Group 1a (n = 19)**	**Group 1b (n = 21)**	**Group 2 (n = 8)**	**Group 3 (n = 13)**	**Group 4 (n = 10)**	**Group 5 (n = 8)**	**Group 6 (n = 13)**
**Packed Cell Volume (%)**		13.6 ± 4.8	17.9 ± 4.3	25.0 ± 14.7	46.8 ± 9.9	44.1 ± 11.8	30.3 ± 15.1	46.9 ± 3.6
		(7.6-23)	(7.0-25.5)	(11.0-55.2)	(18.2-57.7)	(20.5-59.4)	(14.9-61.4)	(41.7-53.2)
**Mean Corpuscular Volume (fl)**		76.7 ± 5.1	77.7 ± 9.3	57.1 ± 3.3	68.9 ± 8.1	70.4 ± 4.5	66.5 ± 5.4	68.7 ± 2.4
		(70.7-88.4)	(67.1-96.3)	(50.4-60.0)	(62.7-77.2)	(62.9-77.3)	(60.0-76.7)	(63.0-71.2)
**Cholesterol concentration (mmol/l)**		6.73 ± 1.37	4.68 ± 3.18	3.58 ± 1.83	16.6 ± 11.0	4.95 ± 1.44	6.33 ± 1.48	6.81 ± 1.45
		(5.3-8.53)*	(0.83-12.7)**	(2.48-8.35)	(6.26-48.38)	(2.77-7.11)	(4.0-9.0)	(4.42-9.15)
**Triglyceride Concentration (mmol/l)**		0.87 ± 0.30	0.76 ± 0.28	0.71 ± 0.44	11.73 ± 9.36	0.75 ± 0.31	0.90 ± 0.34	0.89 ± 0.56
		(0.57-1.25)*	(0.41-1.19)**	(0.24-1.72)	(0.86-29.26)	(0.44-1.34)	(0.52-1.56)	(0.31-1.99)
**Number of spherocytes per high power field**	<1	3	13	7	11	10	7	13
	1-3	2	4	1	2	0	0	0
	3-5	2	3	0	0	0	0	0
	≥5	12	1	0	0	0	1	0
**In-saline auto-agglutination**	Positive	16	0	0	0	0	0	0
	Negative	3	21	8	13	10	8	13
**Coombs’ test**	Positive	13	1	0	0	0	2	0
	Negative	4***	20	8	13	10	6	13

The data of the packed cell volume, the mean corpuscular volume, the cholesterol and triglyceride concentration are expressed as mean ± standard deviation (range).

*Total serum cholesterol and triglyceride concentrations were measured in 4/19 group 1a dogs.

**Total serum cholesterol and triglyceride concentrations were measured in 13/21 group 1b dogs.

***Coombs’ test was performed in 17/19 group 1a dogs.

The results of the in-saline auto-agglutination and Coombs’ tests are summarized in Table 3. Three dogs with IMHA had both a negative in-saline auto-agglutination as well as Coombs’ test. IMHA was diagnosed in these dogs based on the presence of hyperbilirubinaemia, bilirubinuria, a moderate (n = 2) to marked (n = 1) spherocytosis and the absence of another cause of anaemia. In group 1a, the Coombs’ test was not performed in two dogs because of unavailability of a sufficient volume of blood. These dogs were diagnosed with IMHA based upon the presence of haemolytic anaemia with a mild (n = 1) or marked spherocytosis (n = 1) together with a positive in-saline auto-agglutination test. The group 1b dog with a positive Coombs’ test showed Heinz bodies without spherocytes on the blood smear and was diagnosed with methemoglobinaemia due to an onion intoxication. Two dogs from group 5 had a positive Coombs’ test, due to Ehrlichiosis and Leishmaniosis. IMHA was excluded because no signs of haemolytic anaemia were present and all other tests for IMHA were negative.

Table 4 presents the ROFT and COFT results.

**Table 4:** Results of the rapid osmotic fragility test (ROFT) and classic osmotic fragility test (COFT) for group 1a (IMHA), group 1b (anaemia, no IMHA), group 2 (microcytosis), group 3 (hyperlipidemia), group 4 (lymphoma), group 5 (infections) and group 6 (healthy)

.		**Group 1a (n = 19)**	**Group 1b (n = 21)**	**Group 2 (n = 8)**	**Group 3 (n = 13)**	**Group 4 (n = 10)**	**Group 5 (n = 8)**	**Group 6 (n = 13)**
**ROFT**	**Positive**	17	1	0	10	0	0	1
	**Negative**	2	20	8	3	10	8	12
**COFT**	**OF5**	0.75 ± 0.11*	0.51 ± 0.11	0.53 ± 0.13	0.64 ± 0.14*	0.52 ± 0.06	0.49 ± 0.04	0.51 ± 0.04
	**(%)**	(0.51-0.85)	(0.37-0.83)	(0.43-0.81)	(0.43-0.84)	(0.44-0.66)	(0.44-0.52)	(0.46-0.54)
	**OF50**	0.64 ± 0.12*	0.39 ± 0.06	0.39 ± 0.05	0.45 ± 0.05	0.42 ± 0.04	0.39 ± 0.04	0.44 ± 0.03
	**(%)**	(0.44-0.83)	(0.28-0.52)	(0.35-0.49)	(0.33-0.53)	(0.36-0.47)	(0.34-0.42)	(0.40-0.49)
	**OF90**	0.38 ± 0.17	0.27 ± 0.10	0.26 ± 0.09	0.34 ± 0.07	0.36 ± 0.04	0.27 ± 0.10	0.34 ± 0.09
	**(%)**	(0.28-0.64)	(0.06-0.43)	(0.10-0.36)	(0.18-0.40)	(0.31-0.45)	(0.07-0.35)	(0.06-0.40)

Data of the COFT are expressed as mean ± standard deviation (range).

OF5, OF50, OF90 are the NaCl concentration at which respectively 5, 50 and 90% of RBCs are hemolysed.

OF5, OF50 or OF90 from all clinical groups (group 1–5) were compared with the control group (group 6). The global significance level was set at 0.05.

*Denotes a statistically significant difference from the control group for the respective response variable.

Overall, the ROFT was positive in 17/19 (89.4%) of dogs with IMHA and in 12/73 (16.4%) of dogs without IMHA. The two dogs of group 1a with a negative ROFT had primary IMHA and the lowest OF5 and OF50 values of their group. In both dogs, no spherocytes were seen on the blood smear. From the dogs with IMHA with a positive ROFT only one dog had no spherocytosis, while the other dogs had mild (n = 2), moderate (n = 2) or marked (n = 12) spherocytosis. The colour change was very obvious in all 17 dogs from group 1a with a positive ROFT, but was only mildly discernible in the one dog from group 1b with a positive ROFT. This dog, which had anaemia due to chronic kidney disease (International Renal Interest Society stage III), had no spherocytosis. The ROFT was positive in 10/13 (77%) dogs of group 3, with an obvious colour change in seven dogs and a mild colour change in 3 dogs. Two dogs had mild spherocytosis and eight dogs had no spherocytosis. Two of them had only hypercholesterolemia, three only hypertriglyceridemia and five dogs had both hypercholesterolemia and hypertriglyceridemia. In the majority of group 3 dogs, the serum was opaque, and a cloudy supernatant was detected in both ROFT tubes after centrifugation. Finally, although the colour change was very mild, the ROFT was considered positive in one healthy dog (group 6), with a serum TG concentration that approached the upper reference limit. This dog did not have spherocytosis and did not have higher values for OF5, OF50 and OF90 compared to the rest of his group. After centrifugation, the supernatant was cloudy in both ROFT tubes.

### Statistical analysis

**Figure 1:**
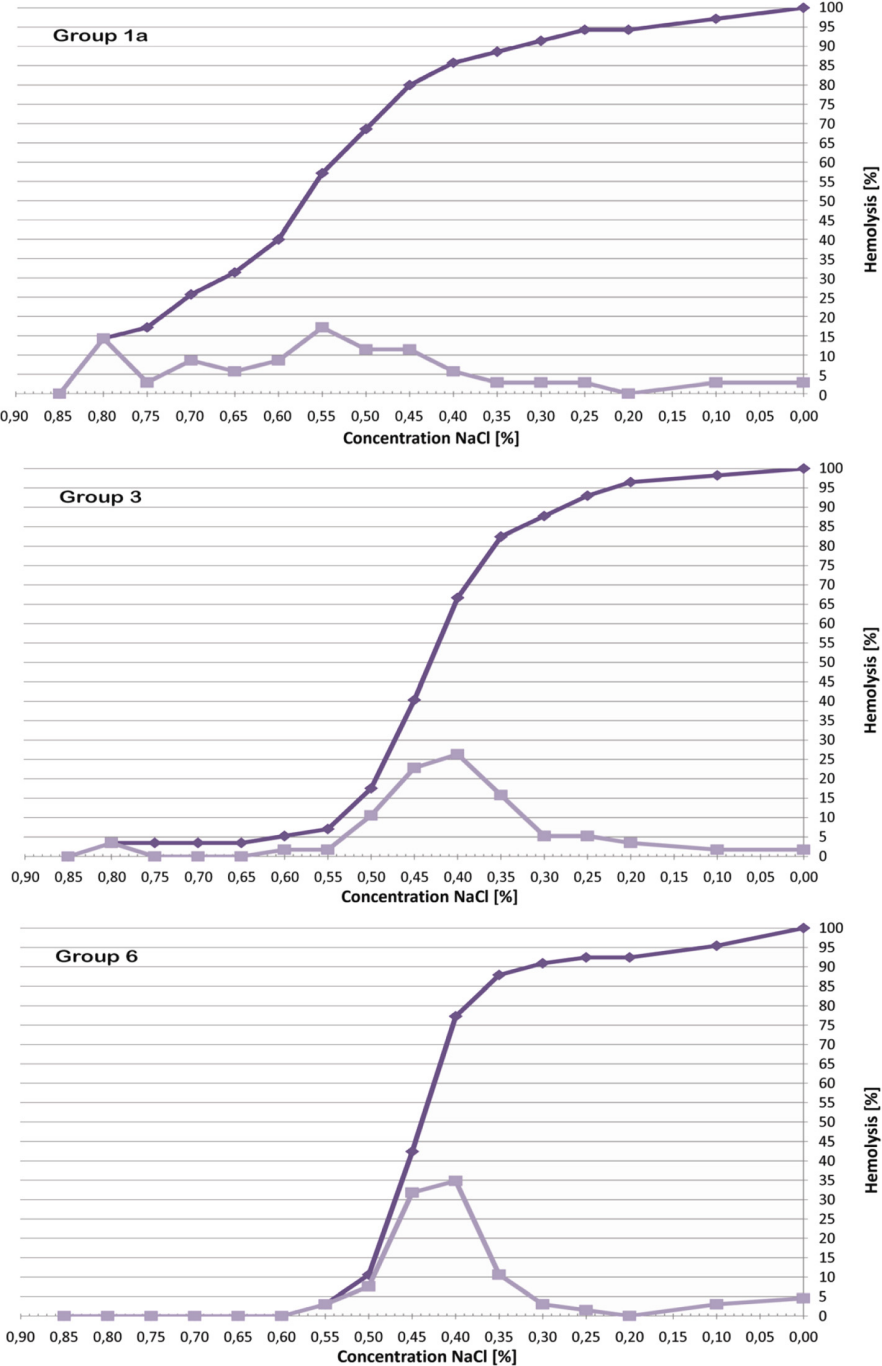
**Cumulative and derivative fragiligram for group 1a (IMHA), group 3 (hyperlipidemia) and group 6 (healthy dogs).** The cumulative (dark line) and derivative (light line) fragiligrams are based on the mean values for OF5, OF50 and OF90 of group 1a (dogs with IMHA), group 3 (dogs with hyperlipidemia) and group 6 (healthy dogs) in the COFT. The figure shows a marked left shift of the cumulative and derivative fragiligram in dogs from group 1a, while no obvious left shift of the fragiligrams is present in dogs from group 3 or group 6. Furthermore, it can be noticed that the NaCl concentration at which 5% of the erythrocytes are haemolysed is higher for group 3 than for group 6, which indicates that in group 3 a small subpopulation of erythrocytes has an increased osmotic fragility.

Compared to the healthy dogs of group 6, OF5 and OF50 of group 1a (*P* < 0.001) and OF5 of group 3 dogs (*P* = 0.0266) were significantly higher. The other comparisons of OF5, OF50 and OF90 among groups did not reveal statistically significant differences. The higher values for OF5 and OF50 in group 1a dogs resulted in a marked left shift of the cumulative and derivative fragiligram. Only two dogs of group 3, those with the highest values of OF5 and OF50 within their group, and none of the others had a left shift of their fragiligrams. Figure 1 depicts the fragiligrams obtained based on the mean values for OF5, OF50 and OF90 of group 1a (IMHA), group 3 (hyperlipidemia) and group 6 (healthy dogs).

Furthermore, it was found that dogs with a positive ROFT had a significant lower PCV (*P* = 0.041), higher MCV (*P* = 0.005), higher number of spherocytes (*P* < 0.001), higher Chol (*P* < 0.001) and higher TG concentrations (*P* < 0.001). Also, a significant positive correlation was found between the number of spherocytes and the OF50 (*r*: 63%; *P* < 0.001).

In comparison, dogs with a positive Coombs’ test had a significantly lower PCV (*P* < 0.001) and a higher number of spherocytes (*P* < 0.001), while MCV (*P* = 0.100), Chol (*P* = 0.831) and TG concentrations (*P* = 0.452) were not significantly different between dogs with a positive Coombs and a negative Coombs’ test. Dogs with a positive Coombs’ test and dogs with a positive in-saline auto-agglutination test also had a significantly higher OF50 (*P* < 0.001).

For group 1, there was no significant difference in the number of reticulocytes between dogs with a positive ROFT and dogs with a negative ROFT (*P* = 0.874). Furthermore, there was no significant difference for OF5 (*P =* 0.228), OF50 (*P* = 0.234) or OF90 (*P* = 0.360) between dogs with regenerative anaemia and dogs with non regenerative anaemia.

The sensitivity and specificity for the ROFT for diagnosing IMHA in dogs with anaemia were respectively 89% and 95%.

## Discussion

Diagnosing canine IMHA is often challenging because all currently available tests have their limitations [1,2]. In human medicine two criteria must be met to diagnose immune-mediated haemolytic anaemia: serological evidence of an autoantibody by positive Coombs’ test and clinical and/or laboratory evidence of haemolysis [29]. In veterinary medicine, diagnosis is similar, but as there is no accepted ‘gold standard’, diagnosis is based upon the presence of haemolytic anaemia in combination with the demonstration of spherocytosis, positive in-saline auto-agglutination and/or Coombs’ test [2].

This study evaluated the OF in dogs with and without IMHA, mainly to assess the diagnostic value of the ROFT as additional test to detect canine IMHA.

A significant increase in OF5 and OF50 and a left shift of the fragiligrams were observed in dogs with IMHA. More importantly, the ROFT was positive in 89% of dogs with IMHA. Few studies evaluated OF in anaemic dogs. Paltrinieri *et al.*[30] used the COFT in 25 healthy and 40 anaemic dogs and found a significant increase in OF5 in dogs with regenerative anaemia (9/15 with haemolytic anaemia) compared to healthy dogs and dogs with non regenerative anaemia. However, they did not specify how many dogs with haemolytic anaemia had immune-mediated disease. In this study, we did not find a significant difference in OF5, OF50 and OF90 between dogs with regenerative and dogs with non regenerative anaemia. Furthermore, the number of reticulocytes was not significantly different between anaemic dogs with a positive ROFT and anaemic dogs with a negative ROFT. To the authors’ knowledge, this study is the first to report the results of COFT and ROFT in a group of dogs with IMHA.

A significant increase in OF5 was also found in dogs with natural occurring hyperlipidemia and 77% of them had a positive ROFT. Furthermore, dogs with a positive ROFT had a significantly higher serum cholesterol and triglyceride concentration. However, in contrast with the IMHA dogs, OF50 was not significantly higher in hyperlipidemic dogs compared with healthy dogs and only 2/13 hyperlipidemic dogs had a left shift of their fragiligrams. This indicates that only a minor subpopulation of RBCs is responsible for the increased OF in the dogs with hyperlipidemia. Previously, hypercholesterolemia, experimentally induced by feeding healthy dogs a cholesterol-enriched atherogenic diet resulted in an increased OF, a decreased PCV and altered RBC morphology after 6 weeks [19]. Although not yet reported in dogs, hypertriglyceridemia can have similar effects and was reported to increase OF without changing the PCV in mice and humans [21,22]. Our study is the first reporting an increased OF in dogs with natural occurring hyperlipidemia. In contrast to the dogs with experimentally induced hypercholesterolemia, none of the dogs of our study with hyperlipidemia developed anaemia, nor showed changes in RBC morphology. Further research is needed to elucidate why not all dogs with hypercholesterolemia and/or hypertriglyceridemia have an increased OF, what the role of the underlying disease process is and what the exact mechanisms are for the increased OF in dogs with natural occurring hyperlipidemia.

Changes in OF were not detected in dogs with microcytosis, lymphoma or infections, in contrast to previous studies for dogs with microcytosis [16,17]. The latter reported an increased OF, with a mild left shift of their fragiligrams, in dogs with a decreased MCV due to internal parasitism. Remarkably, we found that dogs with a positive ROFT had a significant higher MCV (*P* = 0.005) than dogs with a negative ROFT. However, in our study, no dogs had internal parasitism and only a limited number with microcytosis was included, which limits our conclusions about OF and microcytosis in dogs. The influence of neoplasia and infections has yet to be reported. These disease groups were included because previous studies have detected RBC bound antibodies in dogs with neoplasia and dogs that were diagnosed with various infectious diseases without any other additional criteria for the diagnosis of IMHA [1,31]. The clinical relevance of detecting RBC antibodies in non-anaemic dogs is unclear at presence [1]. In our study, the Coombs’ test was positive in two dogs with an infection without signs of haemolytic anaemia. Both dogs had a normal COFT and a negative ROFT. However, because the group of dogs with infections was very heterogeneous and only a small number of dogs with infections was included it is difficult to draw conclusions about OF in dogs with infections based on this study. Further research evaluating OF in dogs with specific infectious conditions is warranted.

The dogs with an increased OF did not belong to one of the breeds in which hereditary erythrocyte membrane defects that can cause an increased OF have been described [7-15]. However, we cannot completely rule out an erythrocyte membrane defect as the cause for the increased OF in these dogs, as, although not yet reported, erythrocyte membrane defects may probably also occur in other breeds. However, in our opinion, it is very unlikely that an erythrocyte membrane defect was the sole cause of the increased OF in the dogs of our study, because all dogs with an increased OF had additional criteria for immune-mediated red cell destruction or had pronounced hyperlipidemia.

Overall, the COFT and ROFT showed similar results in our study population. The ROFT was only positive in dogs with increased OF5 and/or OF50. While the COFT is labour-intensive, the ROFT is a rapid test that can be performed easily in daily practice. However, the COFT provides more detailed information about the OF. For example, the majority of dogs with hyperlipidemia had a positive ROFT, indicating that OF was increased, while the COFT revealed that only OF5 was significantly increased and that, with the exception of two dogs, no left shift of the fragiligrams was present in hyperlipidemic dogs. This was in contrast with what was found for dogs with IMHA, that showed an increase in OF5 and OF50 together with an obvious left shift of their fragiligrams.

A remarkable finding of this study was that the ROFT had a high sensitivity (89%) to diagnose IMHA in dogs in this study. This is an important finding, because a low sensitivity has been reported for some of the other diagnostic tests that are used to diagnose IMHA in dogs, such as the Coombs’ test. The sensitivity of the Coombs’ test has been reported to range between 37 and 89 per cent, but is generally considered to be in the region of 60 per cent [1,32-35]. Also in human medicine ‘Coombs negative auto-immune haemolytic anaemia’ is reported in 1-10% of patients [36]. The large range in sensitivity of the Coombs’ test can be explained because there is no standardized set up of the Coombs’ test in different laboratories [2]. In this study, a commercial available gel-based direct Coombs’ test was used, that has good agreement with the traditional direct Coombs’ test [28]. However, false negatives can occur with the gel-based test. Furthermore, it is reported that the Coombs’ test can quickly become negative after starting treatment with immunosuppressive dosages of corticosteroids and that this can already occur after one day of treatment [28]. In this study, dogs were only excluded if they received medication for longer than 3 days prior to admission. However, only four anaemic dogs received corticosteroids and they all had a positive Coombs’ test. As the sensitivity of the ROFT in our study was only based on a small number of dogs, the results need to be interpreted with caution.

Furthermore, the ROFT had a high specificity (95%) for diagnosing IMHA in anaemic dogs. To accurately determine the specificity of a test, the test should be evaluated in a large population of randomly selected sick and non-sick dogs. However, certain patient groups in our study were included because previous studies documented abnormal OF test results in dogs with these diseases, which resulted in a population of sick dogs that was not randomly selected. Therefore, we could only calculate the specificity of the ROFT for diagnosing IMHA in the anaemic dogs (group 1a and 1b). Further studies are needed to determine the overall specificity of the ROFT and the positive and negative predictive value of this test.

Although, based on the results of this study, the ROFT appeared to be a valuable and practical additional test for diagnosing IMHA in dogs with anaemia, the test has some disadvantages. As it has been assumed that the ROFT is based on the presence of spherocytes and because a moderate to marked spherocytosis is only present in 75 to 94% of dogs with IMHA, false negative results may be expected [35,37,38]. However, although we found that dogs with a positive ROFT had a higher number of spherocytes on their blood smear than dogs with a negative ROFT and that a positive correlation was present between the number of spherocytes and the OF50, it was seen that from the 17 dogs with IMHA and a positive ROFT, one dog had no spherocytosis and two dogs had only mild spherocytosis. In accordance with this, a previous study found spherocytes in 77 of 149 dogs with IMHA, while the OF test, that was performed in 142 of these dogs, was positive in 117 dogs [4]. It is possible that the ROFT has a higher sensitivity for IMHA because low to moderate numbers of spherocytes may not be detected on the blood smear. However, it is more likely that factors other than the presence of spherocytes play a role in the development of the increased OF in dogs with IMHA. It has been reported that in dogs with IMHA a state of oxidative stress and a reduced antioxidant reserve is present [39]. Oxidative stress is thought to aggravate the symptoms of many diseases, including haemolytic anaemias, because erythrocytes are highly susceptible to oxidative damage induced by free radicals or reactive oxygen species [40,41]. Studies in goats, pigs and donkeys showed that stressful conditions such as transportation and packing induced a significant increase in the erythrocyte OF that was not present when the animals were treated with an anti-oxidant (ascorbic acid) before undergoing the stressful event, suggesting that the increased OF was a consequence of oxidative stress [41-43]. Although, not yet studied, oxidative stress may have similar effects on canine erythrocytes and may contribute to the increased OF that is seen in dogs with IMHA. Furthermore, dogs with IMHA are reported to have activated platelets that produce prostaglandin E2 (PGE2) [38]. In humans, it has been reported that PGE2 reduces erythrocyte filterability by stimulating potassium efflux leading to a loss of osmotic water and cell shrinkage [44]. Although, it might be expected that these erythrocytes with a lower volume to surface ratio have an increased OF, it was found that the erythrocyte OF was decreased. Further studies are necessary to evaluate if the effect of PGE2 on canine erythrocyte OF is similar.

A second disadvantage of the ROFT is that a dog can be diagnosed incorrectly with IMHA based on a positive result of the ROFT. In this study, 12 dogs that did not have IMHA, had a positive ROFT. However, some differences were present between the dogs with IMHA and the dogs without IMHA that had a positive ROFT. First, the colour change was very mild in one anaemic dog, one control dog and three dogs with hyperlipidemia with a positive ROFT, while in all dogs with IMHA the change was very obvious. Moreover, in the majority of dogs that did not have IMHA, the supernatant in both ROFT tubes was cloudy, due to hyperlipidemia, while it was clear in all dogs with IMHA.

Unfortunately, as with other diagnostic tests for IMHA, such as the Coombs’ test, in-saline auto-agglutination test and presence of spherocytosis, the ROFT cannot distinguish between primary and secondary IMHA. Finally, the quality of the osmotic fragility test may be influenced by environmental and technical factors. Therefore, it is important that the technical conditions of the assay are strictly standardized [45]. Also, the choice of the anticoagulant can affect the results. For this study, we chose heparinised blood, mainly because, in humans, use of EDTA as an anticoagulant has been described to increase the RBC OF [45]. Using heparinised instead of EDTA blood has as disadvantage that a larger amount of blood needs to be taken in patients that may have a life threatening anaemia. Although only 10 drops of blood are needed to perform the ROFT, it might be more interesting to perform the test on EDTA blood. Further research evaluating the ideal anticoagulant in dogs is warranted. Furthermore, although not seen in the dogs in this study, in dogs with severe haemoglobinaemia due to intravascular haemolysis, the supernatant can be red in both ROFT tubes. In these cases the difference in colour between both ROFT tubes might be only mild. Therefore, it can be more difficult to interpret test results in case of severe haemoglobinaemia. Haemoglobinaemia is present in the minority of dogs with IMHA and is caused by immunoglobulin M (IgM)-mediated haemolysis and subsequent intravascular complement activation with intravascular haemolysis [4,29,46]. However, overwhelming complement activation usually is required to produce clinically evident intravascular haemolysis [29]. In contrast with IgM, immunoglobulin G (IgG), which is the most common Ig isotype associated with IMHA in dogs, is a poor activator of the complement system and therefore causes mainly extravascular haemolysis by macrophages in the liver and/or spleen [29]. In this study, the influence of intravascular versus extravascular haemolysis on OF was not studied, because only few dogs showed signs of intravascular haemolysis (haemoglobinaemia and/or haemoglobinuria) and because some dogs with IMHA have both IgG and IgM antibodies which can result in the combination of intravascular and extravascular haemolysis [4,47]. Finally, as it is unclear if in vitro haemolysis has an influence on COFT and ROFT results, the authors advise to perform analysis, when possible, on blood samples that are not haemolytic. Also, at the moment it is not known if the degree of anaemia might influence the results of the OF tests. Further studies evaluating this are necessary.

Although our results are promising, this study has some limitations. Only a limited number of dogs were included. Furthermore, serum cholesterol and TG concentrations were not measured in every anaemic dog because some of these dogs were not fasted and/or not enough blood could be taken from very anaemic dogs. Nevertheless, it seems highly unlikely that dogs with IMHA had a positive ROFT due to hyperlipidemia as their serum was clear, while it was lipemic in the majority of dogs with hyperlipidemia. Also, none of the dogs with IMHA had a disease that is associated with secondary hyperlipidemia and none of the dogs with IMHA in which the cholesterol and TG concentration was not measured, belonged to a breed in which primary hyperlipidemia has been reported [48]. Finally, in this study spherocytosis was quantified microscopically as counts per oil immersion fields. A disadvantage of our grading system is that the number of spherocytes per high power field depends on the haematocrit [2]. Therefore, calculating the percentage of spherocytes might be more reliable. A grading system in which the number of spherocytes as well as the percentage of spherocytes is included has recently been described [49]. In this system spherocytosis is scored from 1+ to 4+. 1+ equals 5–10 spherocytes per oil immersion field (2-4%), 2+ equals 11–50 per oil immersion field (4-20%), 3+ equals 51–150 per oil immersion field (20-60%), and 4+ equals >150 per oil immersion field (>60%). Because no percentages of spherocytes were calculated in our study, it is impossible to compare the results for spherocytosis from our study with the recently described scoring system. In most previous studies only the presence of spherocytes, without grading spherocytosis, was taken into account [4,28,34,46,50]. In one recent study, a grading system for spherocytosis was used, but it was unclear from the report if this was based on counts per high power field or on percentages of spherocytes [51]. To facilitate the comparison of future studies, standardization of grading of spherocytosis is needed in veterinary medicine. In this study, three dogs were included in group 1a, based on the presence of haemolytic anaemia and the presence of a moderate (n = 2) or marked (n = 1) spherocytosis, while the Coombs’ test and in-saline auto-agglutination test were negative. Although Coombs’ negative IMHA is a well-known phenomenon in dogs [1,32-35], we cannot rule out that these dogs, especially the dogs with only a moderate spherocytosis had haemolytic anaemia due to a non-immune-mediated cause. These dogs were not tested for hereditary erythrocyte membrane defects. However, this seems less likely as one of these dogs was 6 years of age at diagnosis and the other dog, which was younger than a year, had a right hind limb abscess with a suspected secondary IMHA. The haemolytic anaemia in the latter dog resolved with appropriate antibiotic therapy.

## Conclusions

In conclusion, OF was increased in the majority of dogs with IMHA and in dogs with hyperlipidemia, but not in dogs with microcytosis, lymphoma or an infection. Although more detailed information was obtained about the OF by using the COFT, the COFT and ROFT gave similar results. The ROFT does not require specialized equipment, is rapid and easy to perform and can be used easily in daily practice. Although, the ROFT cannot replace other diagnostic tests, it may be a valuable additional tool to diagnose IMHA. Further studies are needed to explain the reason for an increased OF in dogs with IMHA. The degree of spherocytosis likely contributes, but other factors may be involved. Finally, studies with larger number of dogs need to reveal the ideal test conditions and test performances. Because of this, the authors conclude that only anaemic dogs with a positive ROFT, characterized by a clear supernatant that is colourless in the first tube and red in the second tube, are highly likely to have IMHA.

## Abbreviations

IMHA: Immune-mediated haemolytic anaemia; OF: Osmotic fragility; COFT: Classic osmotic fragility test; ROFT: Rapid osmotic fragility test; OF5, OF50, OF90: NaCl concentration at which respectively 5, 50 and 90% of the erythrocytes are haemolyzed; RBC: Red blood cell; PCV: Packed cell volume; MCV: Mean corpuscular volume; Chol: Cholesterol; TG: Triglycerides; CBC: Complete blood count; MGG: May-Grünwald Giemsa; HPF: High power field; PBS: Phosphate buffered saline; PGE2: Prostaglandin E2; Ig: Immunoglobulin.

## Competing interests

The authors declare that they have no competing interests.

## Authors’ contributions

GP participated in study design, carried out the field sampling, data collection and analysis and drafted the manuscript. DP participated in the design of the study, in its coordination and helped to draft the manuscript. EM participated in the spectrophotometrical analysis of the blood samples for the COFT. ATK carried out the blood smear analysis. LD performed the statistical analysis. MC participated in patient collection. SD participated in the design of the study, in its coordination and helped to draft the manuscript. All authors read and approved the final manuscript.

## References

[B1] Morley P Mathes M Guth A Dow S Anti-erythrocyte antibodies and disease associations in anemic and nonanemic dogs J Vet Intern Med 2008 22 886 892 10.1111/j.1939-1676.2008.0112.x 18498322

[B2] Piek CJ Canine idiopathic immune-mediated haemolytic anaemia: a review with recommendations for future research Vet Q 2011 31 129 141 10.1080/01652176.2011.604979 22029883

[B3] Carr AP Panciera DL Kidd L Prognostic factors for mortality and thromboembolism in canine immune-mediated hemolytic anemia: a retrospective study of 72 dogs J Vet Intern Med 2002 16 504 509 10.1111/j.1939-1676.2002.tb02378.x 12322697

[B4] Piek CJ Junius G Dekker A Schrauwen E Slappendel RJ Teske E Idiopathic immune-mediated hemolytic anemia: treatment, outcome and prognostic factors in 149 dogs J Vet Intern Med 2008 22 366 373 10.1111/j.1939-1676.2008.0060.x 18346140

[B5] Slappendel RJ Kirk RW Interpretation of tests for immune-mediated blood diseases Kirk’s Current Veterinary Therapy 1986 9 Philadelphia: Saunders WB 498 505

[B6] Jain NC Hematologic techniques, erythrocyte osmotic fragility test Schalm’s Veterinary Hematology 1986 4 Philadelphia: Lea and Febiger 69 71

[B7] Pinkerton PH Fletch SM Brueckner PJ Miller DR Hereditary stomatocytosis with hemolytic anemia in the dog Blood 1974 44 557 567 4278122

[B8] Giger U Harvey JW Hemolysis caused by phosphofructokinase deficiency in English Springer Spaniels: seven cases (1983–1986) J Am Vet Assoc 1987 191 453 459 2958437

[B9] Maggio-Price L Emerson CL Hinds TR Vincenzi FF Hammond WR Hereditary nonspherocytic hemolytic anemia in beagles Am J Vet Res 1988 49 1020 1025 2458689

[B10] Slappendel RL Renooij W Bruijne JJ Normal cations and abnormal membrane lipids in the red blood cells of dogs with familial stomatocytosis-hypertrophic gastritis Blood 1994 84 904 909 8043871

[B11] LeGrange SN Breitschwerdt EB Grindem CB Beutler E Erythrocyte fragility and chronic intermittent pigmenturia in a dog J Am Vet Assoc 1995 206 1002 1006 7768705

[B12] Bonfanti U Comazzi S Paltrinieri S Bertazzolo W Stomatocytosis in 7 related standard Schnauzers Vet Clin Pathol 2004 33 234 239 10.1111/j.1939-165X.2004.tb00379.x 15570561

[B13] Slappendel RL van Zwieten R van Leeuwen M Schneijdenberg TWM Hereditary spectrin deficiency in Golden Retriever dogs J Vet Intern Med 2005 19 187 192 10.1111/j.1939-1676.2005.tb02680.x 15822562

[B14] Gerber K Harvey JW D’Agorne S Wood J Giger U Hemolysis, myopathy, and cardiac disease associated with hereditary phosphofructokinase deficiency in two whippets Vet Clin Pathol 2009 38 46 51 10.1111/j.1939-165X.2008.00089.x 19228357 PMC2692053

[B15] Hillström A Tvedten H Rowe A Giger U Hereditary phosphofructokinase deficiency in wachtelhunds J Am Anim Hosp Assoc 2011 47 145 150 10.5326/JAAHA-MS-5619 21311071 PMC3132506

[B16] Jain NC Osmotic fragility of erythrocytes of dogs and cats in health and in certain haematological disorders Cornell Vet 1973 63 411 423 4782558

[B17] Makinde MO Bobade PA Osmotic fragility of erythrocytes in clinically normal dogs and dogs infected with parasites Res Vet Sci 1994 57 343 348 10.1016/0034-5288(94)90128-7 7871255

[B18] Akuzawa M Matumoto M Okamoto K Nakashima F Shinozaki M Morizono M Hematological, osmotic and scanning electron microscopic study of erythrocytes of dogs given β-acetylphenylhydrazine Vet Pathol 1989 26 70 74 10.1177/030098588902600111 2913706

[B19] Cooper RA Leslie MH Knight D Detweiler DK Red cell cholesterol enrichment and spur cell anemia in dogs fed a cholesterol-enriched, atherogenic diet J Lipid Res 1980 21 1082 1089 7462804

[B20] Meurs I Hoekstra M van Wanrooij EJA Hildebrand RB Kuiper J Kuipers F Hardeman MR Van Berkel TJC Van Eck M HDL cholesterol levels are an important factor for determining the lifespan of erythrocytes Exp Hematol 2005 33 1309 1319 10.1016/j.exphem.2005.07.004 16263415

[B21] Cantin B Boudriau S Bertrand M Brun L Gagné C Rogers PA Ven Murthy MR Lupien P Julien P Hemolysis in primary lipoprotein lipase deficiency Metabolism 1995 44 652 658 10.1016/0026-0495(95)90124-8 7752915

[B22] Zhao T Guo J Li H Huang W Xian X Ross CJD Hayden MR Wen Z Liu G Hemorheological abnormalities in lipoprotein lipase deficient mice with severe hypertriglyceridemia Biochem Biophys Res Commun 2006 341 1066 1071 10.1016/j.bbrc.2006.01.067 16460682

[B23] Gookin JL Bunch SE Rush LJ Grindem CB Evaluation of microcytosis in 18 Shibas J Am Vet Assoc 1998 15 1258 1259 9569165

[B24] Giger U Ettinger JE, Feldman EC Regenerative anemia caused by blood loss or hemolysis Textbook of veterinary internal medicine 2005 7 St. Louis: Elsevier Saunders 1886 1907

[B25] Day MJ Day MJ Immune-mediated haematological disease Clinical immunology of the dog and the cat 2008 2 London: Manson Publishing Ltd 94 120

[B26] Couto CG Nelson RW Anemia Small animal internal medicine 2009 4 St. Louis (MO): Mosby Elsevier 1215

[B27] Lapierre Y Rigal D Adam J Josef D Meyer F Greber S Drot C The gel test: a new way to detect red cell antigen-antibody reactions Transfusion 2012 30 109 113 2305438 10.1046/j.1537-2995.1990.30290162894.x

[B28] Piek CJ Teske E van Leeuwen MW Day MJ Good agreement of conventional and gel-based direct antiglobulin test in immune-mediated haemolytic anaemia Acta Vet Scand 2012 54 10 10.1186/1751-0147-54-10 22316049 PMC3296606

[B29] Gehrs BC Friedberg RC Autoimmune Hemolytic Anemia Am J Hematol 2002 69 258 271 10.1002/ajh.10062 11921020

[B30] Paltrinieri S Comazzi S Agnes F Haematological parameters and altered erythrocyte metabolism in anaemic dogs J Comp Pathol 2000 34 122 125 10.1053/jcpa.1999.0339 10627388

[B31] Slappendel RJ The diagnostic significance of the direct antiglobulin test (DAT) in anemic dogs Vet Immunol Immunopathol 1979 1 49 59 10.1016/0165-2427(79)90007-2 15612269

[B32] Switzer JW Jain NC Autoimmune hemolytic anemia in dogs and cats Vet Clin North Am Small Anim Pract 1981 11 405 420 6976035 10.1016/s0195-5616(81)50036-2

[B33] Slappendel RL Abnormal osmotic fragility of erythrocytes in dogs and cats Vet Q 1998 20 38 39 10.1080/01652176.1998.10807399 9651995

[B34] Reimer ME Troy GC Warnick LD Immune-mediated hemolytic anemia: 70 cases (1988–1996) J Am Anim Hosp Assoc 1999 35 384 391 10493413 10.5326/15473317-35-5-384

[B35] Burgess K Moore A Rand W Cotter SM Treatment of immune-mediated hemolytic anemia in dogs with cyclophosphamide J Vet Intern Med 2000 14 456 462 10.1111/j.1939-1676.2000.tb02256.x 10935898

[B36] Kamesaki T Oyamada T Omine M Ozawa K Kajii E Cut-off value of red-blood-cell-bound IgG for the diagnosis of Coombs-negative autoimmune hemolytic anemia Am J Hematol 2009 84 98 101 10.1002/ajh.21336 19105232

[B37] Mason N Duval D Shofer FS Giger U Cyclophosphamide exerts no beneficial effect over prednisone alone in the initial treatment of acute immune-mediated hemolytic anemia in dogs: a randomized controlled clinical trial J Vet Intern Med 2003 17 206 212 10.1111/j.1939-1676.2003.tb02435.x 12683622

[B38] Weiss DJ Brazzell JL Detection of activated platelets in dogs with primary immune-mediated hemolytic anemia J Vet Intern Med 2006 20 682 686 10.1111/j.1939-1676.2006.tb02915.x 16734108

[B39] Pesillo SA Freeman LM Rush JE Assessment of lipid peroxidation ans serum vitamin E concentration in dogs with immune-mediated hemolytic anemia Am J Vet Res 2004 65 1621 1624 10.2460/ajvr.2004.65.1621 15631024

[B40] Fibach E Rachmilewitz E The role of oxidative stress in hemolytic anemia Curr Mol Med 2008 8 609 619 10.2174/156652408786241384 18991647

[B41] Minka NS Ayo JO Physiological response of erythrocytes of goats to transportation and the mondulatory role of ascorbic acid J Vet Med Sci 2012 72 875 881 20203435 10.1292/jvms.09-0531

[B42] Asala OO Ayo JO Rekwot PI Minka NS Omoniwa DO Adenkola AY Effect of ascorbic acid administration on erythrocyte osmotic fragility of pigs transported by road during the hot-dry season Vet Res Commun 2011 35 245 254 10.1007/s11259-011-9469-7 21347678

[B43] Olaifa F Ayo JO Ambali SF Rekwot PI Effect of packing on changes in erythrocyte osmotic fragility and malondialdehyde concentration in donkeys administered with ascorbic acid J Vet Res 2012 79 1 5 10.4102/ojvr.v79i1.413 23327322

[B44] Li Q Jungmann V Kiyatkin A Low PS Prostaglandin E_2_ stimulates a Ca^2+^-dependent K^+^ channel in human erythrocytes and alters cell volume and filterability J Biol Chem 1996 271 18651 18656 10.1074/jbc.271.31.18651 8702518

[B45] Kafka M Yermiahu T The effect of EDTA as an anticoagulant on the osmotic fragility of erythrocytes Clin Haematol Lab 1998 20 213 216 10.1046/j.1365-2257.1998.00014.x 9777266

[B46] Klag AR Giger U Shofer FS Idiopathic immune-mediated hemolytic anemia in dogs: 42 cases (1986–1990) J Am Vet Med Assoc 1993 202 783 788 8454517

[B47] Stockham SL Scott MA Erythrocytes Fundamentals of Veterinary Clinical Pathology 2008 2 Blackwell Publishing Professional 170 176

[B48] Xenoulis PG Steiner JM Lipid metabolism and hyperlipidemia in dogs Vet J 2010 1 12 21 19167915 10.1016/j.tvjl.2008.10.011

[B49] Willard M Tvedten H Willard MD, Tvedten H The complete blood count, bone marrow examination, and blood banking: general comments and selected techniques Small animal clinical diagnosis by laboratory methods 2012 5 St. Louis (MO): Elsevier Saunders 29

[B50] Weinkle TK Center SA Randolph JF Warner KL Carr SC Erb HN Evaluation of prognostic factors, survival rates, and treatment protocols for immune-mediated hemolytic anemia in dogs: 151 cases (1993–2002) J Am Vet Med Assoc 2005 11 1869 1880 15934255 10.2460/javma.2005.226.1869

[B51] Harkin KR Hicks JA Wilkerson MJ Erythrocyte-bound immunoglobulin isotypes in dogs with immune-mediated hemolytic anemia: 54 cases (2001–2010) J Am Vet Med Assoc 2012 241 227 232 10.2460/javma.241.2.227 22765369

